# Microbiota and Recurrent Pregnancy Loss (RPL); More than a Simple Connection

**DOI:** 10.3390/microorganisms12081641

**Published:** 2024-08-10

**Authors:** Jenny Valentina Garmendia, Claudia Valentina De Sanctis, Marián Hajdúch, Juan Bautista De Sanctis

**Affiliations:** 1 Institute of Molecular and Translational Medicine, Faculty of Medicine and Dentistry, Palacky University, Hněvotínská 1333/5, 779 00 Olomouc, Czech Republic; jenny.garmendia@gmail.com (J.V.G.); marian.hajduch@upol.cz (M.H.); 2 Czech Advanced Technology and Research Institute, Palacky University, 779 00 Olomouc, Czech Republic; 3 Laboratory of Experimental Medicine, University Hospital Olomouc (FNOL), Faculty of Medicine and Dentistry, Palacky University, 779 00 Olomouc, Czech Republic

**Keywords:** recurrent pregnancy loss (RPL), recurrent implantation failure (RIF), vaginal microbiota, uterine microbiota, dysbiosis, probiotic supplementation, bacterial transplantation

## Abstract

Recurrent Pregnancy Loss (RPL) affects 1–2% of women, and its triggering factors are unclear. Several studies have shown that the vaginal, endometrial, and gut microbiota may play a role in RPL. A decrease in the quantity of *Lactobacillus crispatus* in local microbiota has been associated with an increase in local (vaginal and endometrial) inflammatory response and immune cell activation that leads to pregnancy loss. The inflammatory response may be triggered by gram-negative bacteria, lipopolysaccharides (LPS), viral infections, mycosis, or atypia (tumor growth). Bacterial structures and metabolites produced by microbiota could be involved in immune cell modulation and may be responsible for immune cell activation and molecular mimicry. Gut microbiota metabolic products may increase the amount of circulating pro-inflammatory lymphocytes, which, in turn, will migrate into vaginal or endometrial tissues. Local pro-inflammatory Th1 and Th17 subpopulations and a decrease in local Treg and tolerogenic NK cells are accountable for the increase in pregnancy loss. Local microbiota may modulate the local inflammatory response, increasing pregnancy success. Analyzing local and gut microbiota may be necessary to characterize some RPL patients. Although oral supplementation of probiotics has not been shown to modify vaginal or endometrial microbiota, the metabolites produced by it may benefit patients. *Lactobacillus crispatus* transplantation into the vagina may enhance the required immune tolerogenic response to achieve a normal pregnancy. The effect of hormone stimulation and progesterone to maintain early pregnancy on microbiota has not been adequately studied, and more research is needed in this area. Well-designed clinical trials are required to ascertain the benefit of microbiota modulation in RPL.

## 1. Introduction

Recurrent pregnancy loss (RPL) is defined as the pregnancy loss before 20 weeks of gestation of two (US) or three (UK) consecutive clinical pregnancies. The loss must be documented by ultrasound or histopathology. This condition affects 1–2% of women of reproductive age [1,2,3] and is linked to genetic, anatomical, endocrine, autoimmune, and infectious factors [1,3]. Most cases of RPL lack clear etiology, and little is known about its associated factors [1,2,3]. 

There are two types of RPL: primary and secondary [3]. Primary RPL occurs in women who have never given birth to a live infant, while secondary RPL occurs in women who have given birth to a live infant. More research is required to define the differences between the two entities. It is generally assumed that the mechanisms of primary and secondary RPL differ, but this has yet to be proven.

Great efforts have been made to analyze vaginal and endometrial/uterine conditions in RPL [3]. The analysis of local tissue along with resident immune cells and the plausible role of infectious diseases in modifying local homeostasis has rendered exciting results [4,5,6]. Except for explainable problems with the spermatozoa, low amount, low migration, impaired function, and genetic mutations of the zygote/fetus, the other reason for RPL mainly involves maternal physiological responses. In primary RPL, there is a need to understand the key elements that are involved in the increase of recurrent abortion; in secondary RPL, there is a need to know why, after a pregnancy, it has been impossible to achieve another normal pregnancy. Local factors may play a critical role in the process. 

Microbiota are the diverse microbial communities home in the human body [4,5,6]. This comprises bacteria, archaebacteria, fungi, viruses, and protists. These communities vary significantly in composition and function among different body sites and individuals. *Lactobacilli* dominate normal vaginal and endometrial microbiota. Local normal microbiota plays a role in the defence of external pathogens and the modulation of local immune response [4,5,6,7]. Dysbiosis refers to an imbalance or maladaptation of bacterial communities. The vaginal microbiota is not stable and can fluctuate throughout a woman’s life cycle and during her menstrual cycle. Vaginal dysbiosis is identified as a microbiota that is not dominated by *Lactobacillus* spp. This dysbiotic, lactobacilli-depleted vaginal microbiota has been linked to increased susceptibility to sexually transmitted infections and an elevated risk of pregnancy complications [4]. Two key elements have been defined: the importance of zygote implantation and immune tolerogenic response to avoid fetal rejection [3,4,5,6,7]. In both permissive conditions, local microbiota plays a critical role against foreign pathogens, protecting tissues and promoting a vigilant response from local immune cells.

This brief overview will analyze local microbiota, the modulation of local and gut microbiota, and its relationship with local immune response. It will also discuss new and exciting strategies to facilitate physiological and therapeutic responses.

## 2. Local Microbiota

The microbial population that lives in the vagina is composed of facultative and obligate anaerobes that form a symbiotic relationship with the host [8]. These bacteria maintain healthy vaginal tissue by promoting and supporting an ideal pH of ~4, producing hydrogen peroxide [8] and a proteinaceous outer protective layer [8,9,10]. Different species of *Lactobacillus* appear to be most prevalent among women [8,9,10,11], with *L.  crispatus being* categorized as the most protective species and found in the highest proportions in healthy individuals who had successful pregnancies and *L. iners* being the least protective. It is unclear how different *Lactobacillus* species affect zygote implantation or pregnancy progression [8,9,10,11]. 

In vaginal dysbiosis, there is a remarkable decrease in *L. * crispatus with the concomitant increase in *Gardnerella* spp., *Prevotella* spp., *Mobilincus* spp., *Megaspahera* spp., *Sneathea* spp., and mixed vaginal anaerobes species [12,13]. In addition, the presence of *Propionibacterium* spp., *Eubacterium* spp., *Peptostreptococcus* spp., *Bacteroides* spp., *Prevotella* spp., *Porphyromonas* spp., *Fusobacterium* spp., *Veillonella* spp., *Corynebacterium* spp., *Staphylococcus* spp., *Streptococcus* spp., *Enterococcus* spp., *Enterobacter* spp., *E. coli*, *Klebsiella* spp., and *Gardnerella vaginalis* in the endometrium is associated with bacterial vaginosis responsible the inflammatory conditions unsuitable for zygote implantation [13,14,15]. 

The local microbiota in the female reproductive organs is complex and modulated by several factors. Microbiota plasticity, the ability to adapt its composition to align with the needs of the host, occurs during the menstrual cycle. The bacterial abundance changes during specific menstrual cycle phases and may lead to confusing results. During menstruation, there is an increase in *Gardnerella* spp. or *L. iners*, *Prevotella* spp., and *Sneathia* spp. with a decrease in *L. crispatus* [16,17,18]. However, protective *Lactobacillus* species increase to the highest during the luteal phase [16,17,18]. The presence of *Prevotella* spp. is linked to the proliferative phase of the menstrual cycle, whereas *Sneathia* spp. is related to the secretory phase [17,18]. Metabolic activity is crucial in communicating between the host and microbiota in the receptive phase endometrium, particularly in the prostanoid biosynthesis pathway and L-tryptophan metabolism [6,8,19]. Local microbiota can be affected by the host metabolic conditions: overweight, underweight, endocrine disarrangements, and some non-manifested conditions (subclinical inflammation or autoimmunity) [6,19,20]. Therefore, the microbiota is responsive to the host’s conditions and environmental, hormonal, and dietary changes [8,19,20]. 

Local microbiota changes can occur in sexually active women, and these alterations may be dependent on sperm microbiota [21,22]. A dysbiotic microbiota is less protective against sexually and non-sexually transmitted diseases such as HIV [23,24], syphilis, chlamydia, gonorrhea, Trichomonas [23,24], human papillomavirus (HPV) [25,26,27], herpes simplex virus (HSV) [28], pelvic inflammatory disease [29,30], aerobic vaginitis (AV) [29,30], bacterial vaginosis (BV) [29,30] and candidiasis [30,31,32]; all of which can negatively affect gestation [29,30,31,32] by causing inflammation and tissue destruction [33]. HPV can induce essential changes in the local microbiota; viral infection alters the local secretion of IFNs type I and III, activating the immune system [25,26,27]. In addition, patients with persistent HPV infection had significantly higher levels of *Bacteroidaceae*, *Erysipelotrichaceae*, *Helicobacteraceae*, *Neisseriaceae*, *Streptococcaceae* (family level), and *Fusobacterium*, *Bacteroides*, *Neisseria*, and *Helicobacter* (genus level) than patients who had cleared HPV suggesting that the microbiota may be involved in antiviral immune response [25,26,27]. *L. gasseri* LGV03, isolated from the cervical fluid of patients, is indirectly involved in virus clearance, keeps the innate system alert to potential pathogens, and reduces the inflammatory effects during persistent pathogen infection [34]. Conversely, changes in the vaginal, endometrial, and gut microbiota are influenced by the inflammatory response triggered by the abnormal growth of the endometrial tissue in endometriosis.

Interestingly, the use of hormonal contraception has not been shown to impact the microbiota composition in the vagina, feces, rectum, or saliva in healthy young women [35]. This is an important finding, considering the widespread use of these effective contraceptive methods. Conversely, in patients undergoing in vitro fertilization (IVF) procedures, some reports have shown that the use of hormonal therapies before and after the procedure alters vaginal or endometrial microbiota [36,37], while others have not [38]. If vaginal dysbiosis occurs before IVF treatment, the success rate decreases significantly; dysbiosis influences the outcome of the procedure.

It is important to consider other issues in the analysis of microbiota. In animal models, the local microbiota differs from that of humans. As a result, the interpretations of the analysis may need to be revised [39]. Several reports have shown discrepancies in the statistical association between bacterial species, preterm birth, and race [40,41]. This raises questions about possible genetic links between the host, local microbiota, and immune response in RPL.

### Microbiota Recurrent Implantation Failure and Recurrent Pregnancy Loss

Recurrent implantation failure (RIF) and RPL are associated with increased microbiome diversity and a loss of *Lactobacillus crispatus* dominance in the lower female reproductive system [6]. First-trimester miscarriage has been associated with a reduced prevalence of *Lactobacillus crispatus* in vaginal microbiota [42]. The first report by Nelson and coworkers in 2007 identified the importance of the *Lactobacilliae* species in pregnancy loss, confirmed later [43]. Other researchers have reported, in populations of different geographical areas, the role of pathogenic bacteria in vaginosis [43,44,45,46,47,48,49,50,51,52,53,54,55,56,57,58,59,60,61,62,63,64,65,66,67]. Table 1 illustrates a group of reports and the most critical conclusion concerning microbiota and RPL or miscarriage.

Several reports have shown a prevalence of the genera *Ureaplasma*, *Gardnerella*, *Megastrobilia*, *Prevotella*, *Enterococcus*, *Staphylococcus*, and other gram-negative bacteria over *Lactobacillus* spp., and poor microbiota plasticity (little to no changes in the bacterial population in response to different signals) is associated with an increased risk of infections and RPL [43,44,45,46,47,48,49,50,51,52,53,54,55,56,57,58,59,60,61,62,63,64,65,66,67]. *Lactobacillus* spp. Depleted vaginal microbiota was related to the presence of pro-inflammatory cytokine (IL-1β, IL-6, IL-8) levels. This effect is observed most strongly in euploid miscarriage compared to viable term pregnancy [68,69]. On the other hand, *Lactobacillus crispatus* was less abundant in the endometrial samples of women with RPL compared with controls, and *Gardnerella vaginalis* was more abundant in the RPL group than in controls in both endometrial and vaginal samples [69]. 

Vomstein et al. [57,70] observed a lower abundance of the genera *Lactobacillaceae* in the uterus of RPL and RIF patients at three points of their menstrual cycle. They found an increase in the genera *Pseudomonadota* in the RSA and RIF groups towards the end of the menstrual cycle. In this study, the RIF group exhibited a remarkably diverse composition, unlike the control and the RPL group [56]. In the same way, a relative dominance rate of *Ureaplasma* species in the endometrial microbiome was an independent risk factor for subsequent miscarriage with normal karyotype in a cohort of patients with a history of RPL [71]. The genera *Pseudomonadota* and *Bacillota* were significantly elevated in the endometrium of RPL patients in comparison with women requesting termination of normal pregnancy [61]. The abundance of the genera *Bacteroides* and *Helicobacter* in the vagina in an early embryonic arrest group was higher than that in the standard pregnancy-induced abortion group. Furthermore, the abundance of *Lactobacillus crispatus* spp. in the normal pregnancy-induced abortion group was higher than that in the embryonic arrest group. In this last group, the abundance of *L. iners* was significantly lower than that in the normal pregnancy group [72]. Therefore, women with an unbalanced population of bacteria of the genera *Gardnerella*, *Prevotella*, *Atopobium*, *Sneathia*, *Megasphaera*, *Delftia*, *Cutibacterium*, *Peptoniphilus*, *Anaerobacillus* are at higher risk for premature birth and RPL [69,73,74,75,76] than those with the genus *Lactobacillus*. Smith and Ravel [77] proposed a hierarchical clustering of the vaginal microbiota of reproductive-aged women into five distinct community state types (CST), four of which are dominated by *Lactobacillus* spp. (*Lactobacillus crispatus* (CST-I), *L. iners* (CST-III), *L. gasseri* (CST-II) or *L. jensenii* (CST-V)) and the fifth (CST-IV) is composed of a polymicrobial mixture of strict and facultative anaerobes, including species of the genera *Atopobium*, *Megasphera*, *Mobiluncus*, *Prevotella*, and sometimes bacteria of the genera *Eubacteriales*. CST I correlates with low obstetric-gynecological risk, and CST IV correlates mostly with vaginal discomfort and/or obstetric-gynecological diseases [44,45].

The uterine endometrium microbiota (UEM) composition might predict pregnancy outcomes [71]. A dysbiotic UEM, consisting of *Lactobacillus iners* and *Ureaplasma* species, is associated with inflammatory conditions like chronic endometritis (CE) [60], and women with this condition are likely to be diagnosed with RPL, RIF, and infertility [78,79]. CE occurs when plasma cells and B lymphocytes migrate to the uterine endometrial stroma, creating permanent inflammation [80]. The administration of antibiotics has been shown to improve implantation outcomes [81,82] but does not necessarily improve miscarriage rates [81,82]. These results open new questions on the role of bacterial diversity and plasticity in the endometrium.

Changes in local microbiota affect not only observed in RIF or RPL patients but also preeclampsia; an increase in *Escherichia* species was reported along with *Rothia*, *Actinomyces*, and *Enterococcus*, and a lower abundance of *Coprococcus* compared to pregnant women with normotension [83]. Changes also occur in eclampsia and diabetic pregnancies [84,85]. In a study conducted in China [86], the proportion of preterm births was higher in the group with gestational diabetes as compared to the control. There were changes in the vaginal microbiota in the third trimester. *Lactobacillus paragasseri/gasseri*, *Streptococcus* spp., and the genera *Pseudomonadota* were abundant in the preterm birth group [86]. However, *L. mulieris* (a new species of the *L. delbrueckii* group) was associated with a decreased risk of preterm birth [87]. These results suggest that different subspecies of *L. delbrueckii* should be screened in patients with RPL. The newly encountered species could be used in local therapy.

Only a few reports relate RPL to metabolic syndrome after 40 years of age [88]. This association can be due to different events but may involve the gut microbiota. More research is required in this area.

## 3. Impact of Gut Microbiota on Vaginal and Endometrial Microbiota

Gut microbiota has an important metabolic role; it converts food particles into essential nutrients [88,89] and neurotransmitters and helps modulate insulin response. The association between dysbiotic gut microbiota and RPL is complex. Gut microbiota participates in numerous pathologies associated with higher incidences of RLP, such as chronic inflammation, vulnerability to infections, obesity, diabetes, and polycystic ovary syndrome (PCOS) [89,90]. The type of bacterial population in the gut microbiota is involved in local inflammatory responses due to abnormal production of cytokines [91]. 

Several authors have proposed a close relationship between reproductive and gut microbiota [89,90,91,92,93,94,95]. Zhu et al. [92] recently provided evidence suggesting a link between dysbiotic gut microbiota and RPL. The gut microbiota, composed of symbiotic bacteria, is involved in metabolism, inflammation, and immunity [93]. To protect the host from infections, bacteria release factors and form a physical barrier by attaching to the intestinal wall [94]. When the physical protective barrier decreases, cell interaction may be impaired, facilitating the passage of bacteria and toxins into the bloodstream (leaky syndrome) and generating an inflammatory response. The generation of adaptative responses against different antigens from pathogens and toxins may result in autoimmunity due to similarities between typical protein structures and pathogenic proteins. Leaky syndrome, intestinal, vaginal, or endometrial, and most probably vaginal or endometrial, affects reproductive organ microbiota [93,95] and dysbiosis and affects gut microbiota [4,95]. The increase in vaginal and endometrial infection is related to the rise in preterm birth; LPS may be involved as a trigger of the inflammatory response [91,96]. Moreover, LPS is also known to increase the production of reactive oxygen species (ROS), resulting in oxidative stress as well as insulin resistance (IR) [97,98]. 

The vaginal and endometrial abundance of non-protective bacterial species such as *Prevotella* [99] promotes inflammation that leads to immune cell migration and activation [100] and excessive neutrophil, macrophage, and NK cell migration [101]. Autoimmune diseases such as Systemic Lupus Erythematosus (SLE), autoimmune thyroid disease (AITD), and celiac disease (CD) are all associated with higher incidences of RPL and a dysbiotic gut [102,103,104,105,106,107]. It is yet unclear whether treatment with the “beneficial” *Lactobacillus* species (*L. crispatus*) can improve autoimmunity and thus reduce the likelihood of fetal rejection [8,106,107]. 

Yang P and coworkers reported a link between controlled production and activation of NK cells and the reduction of pro-inflammatory cells, even in the placenta [108]. A higher occurrence of *Gardnerella vaginalis* and gram-negative bacteria has been linked to an increased count of circulating NK cells and recurrent miscarriage [4,50], and this effect may be critical in the response of NK cells to estrogen [109]. Interestingly, taking *Bifidobacterium* supplements by mouth has been suggested to improve infertility and reduce microbiota imbalance [110].

## 4. Immune Cells in the Female Reproductive Tract

### 4.1. Innate Immunity

The female reproductive tract has a physical barrier of mucous layer, IgA antibodies, and a commensal microbiota to defend against pathogens. IgA is the protective antibody in dysbiosis [111]. Epithelial cells secrete antimicrobial peptides (AMP) and play a crucial role in protecting against pathogens and regulating immune responses. AMPs are also linked to crucial processes during embryo implantation and pregnancy complications [5,9,49,112]. Macrophages and DCs comprise 10–20% of the local leukocyte population and are responsible for surveilling microbiotas and acting as antigen-presenting cells [5,9]. They have pattern recognition receptors (PRRs) and can recognize microbial signals, initiating a protective immune response. The expression of these receptors decreases during the proliferative phase and increases during the secretory phase. Also, they have danger receptors involved in immune cell activation. Microbial stimulation of PRRs by peptidoglycans, lipoglycans, glycans, and bacterial-secreted proteins leads to the secretion of IL-1β, IL-6, IL-8, and TNF-α, recruiting or activating specialized immune cells [5,9,49,112,113,114,115,116,117,118,119,120,121,122,123,124,125,126,127,128,129,130,131,132,133,134,135,136,137,138,139,140,141,142,143,144,145,146,147,148,149,150,151,152].

Uterine NK cells also play an essential part in pathogen elimination, and decidual NK cells protect the embryo from the harmful effects of infection [152]. The tissue milieu facilitates pathogen elimination, cell migration from peripheral blood, cell priming, successful implantation, and fetal survival.

### 4.2. Adaptative Immunity

Immune cells vary among different parts of the female reproductive tract [7,114,115]. In the vagina, contrary to expectation, T cells (CD4^+^ and CD8^+^ subpopulations, memory cells) predominate at around 50% of the total leucocyte, NK cells are the second highest population, close to 20%, the number of B cells is only 1%, and the rest of the cells, approximately 30%, are granulocytes and macrophages [7,115]. As a comparison, semen contains preferentially granulocytes and macrophages and a small number of lymphocytes (~5%) [116]. 

T cells (CD4^+^ and CD8^+^) are around 50% of the leucocytes in the ecto and endocervix, with macrophages and NK cells representing around 12% [7,115]. The rest are granulocytes and B lymphocytes. Finally, the cell type in the endometrium differs depending on the hormonal cycle. From the early follicular to the early secretory phase, the number of leucocytes (CD45^+^) in the endometrium remains low, but during the secretory phase, they increase about 5-fold [7]. Therefore, the total number of leukocytes peaks premenstrually. In the late secretory phase, NK cells (especially CD3^−^/CD56^bright^/CD16^−^) are predominant, compromising approximately 80% of CD45^+^ cells, while CD3^+^ T cells (predominantly CD8^+^) decrease to less than 10% [7,114,115]. The percentage of B cells is low in the proliferative and middle secretory phases and increases in the late secretory phase [7,115]. Macrophages, neutrophils, and eosinophils increase at the late secretory phase. Mast cells represent 1–2% of the total endometrial cells during the menstrual cycle. T cells are high in the Fallopian tube, followed by granulocytes, NK cells, macrophages, and B cells [7,115]. In summary, in the endometrium, cells have high mobility depending on the hormonal cycle, which may change in case of infection. As expected, the immune cell response will differ according to the type (viral, bacterial, fungal), infection site, and the hormonal cycle stage [7,115]. Immune cells in the sperm are usually Tγδ cells, which contribute to the tolerogenic inhibition of B cells [116]. Rarely are neutrophils present in the semen unless an infection is detected [116]. It is debatable whether immune cells in the semen can alter vaginal microbiota.

Th1 cells are required for zygote implantation, and after that, there is a shift in T cell subpopulations, with Th2 being the predominant local T cell. Th1 cells can be increased in the local milieu upon infection, facilitating zygote implantation. Still, the failure to switch to Th2 is suspected to be the cause of pregnancy loss at early stages [5,7,114,115]. 

It is also important to clarify that mucosal T cells (MAIT) and innate lymphoid cells will migrate to the reproductive tissues in the presence of infection [7,115,117,118,119]. Three types of innate lymphoid cells are precursors of Th1, Th2, and Th17/Th22, depending on the tissue milieu [118]. The role of immune cells, particularly uterine and decidual NK cells, has been reviewed before [114]. 

The role of IFN signaling upon viral infection may condition endometrial local immune response [118,119]. Cell activation by Toll-like receptors or danger cell signals and inflammasome [116] activation negatively impacts RPL [113,114,115,116,117,118,119,120]. The production of local cytokines may partially explain the local response [68,69,121], and semen impacts local cytokine production [122]. 

Pathogens in the female genital tract are typically identified by Toll-like receptors [123,124], triggering the innate immune response. An excessive number of pathogens can lead to over-activation of the innate immune response, making it challenging to resolve chronic inflammation in the reproductive organs [125]. It is unclear if secondary RPL is due to chronic inflammation of the endometrium. The role of immunoglobulins, complement, and antibacterial peptides in local infection resolution requires more research.

Human leukocyte antigen genes (HLA) have been related to oral, intestinal, vaginal, and endometrial microbiota [126,127]. Certain HLA haplotype carriers might be more susceptible to having a dysbiotic microbial population; in fact, women with the HLA-DQ2/DQ8 haplotype appear to have an altered microbiota [65]. In a recent review, Barryman and coworkers [127] have illustrated that microbiota dysbiosis changes occur before the onset of autoimmunity and are linked to HLA. Interestingly, gut *Lactobacillus* and *Bifidobacterium* are considered protective. The link of protective bacteria leads to a hypothesis that a group of RPL women may be at risk of developing an autoimmune disease, and antigen mimicry is responsible for this effect. There are several reports to support the hypothesis.

Around 15% of the patients with RPL have thyroid autoimmunity [128,129], and the HLA alleles associated with RPL patients are linked to autoimmune diseases: DRB1*1501 with multiple sclerosis, DRB1*07 with interstitial lung disease, and DQB1*05 with autoimmune encephalitis. Interestingly, HLA-DRB1*07 has also been linked to lung fibrosis, which can be related to molecular mimicry [130,131]. Thus, gut microbiota dysbiosis may be an early predictor of autoimmunity associated with RPL that has not manifested clinically. 

**Figure 1:**
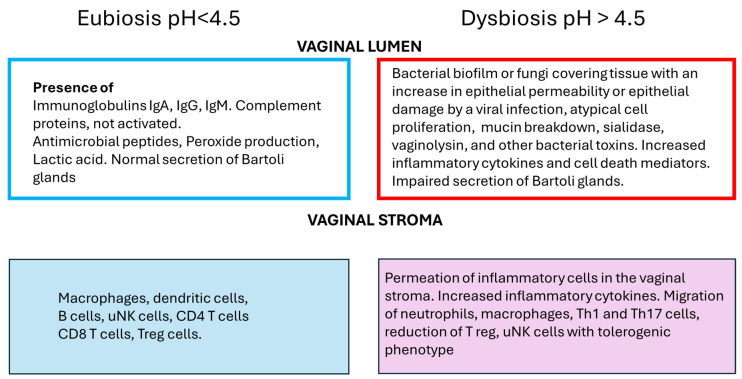
General overview of the differences between eubiosis and dysbiosis in the vagina. In the vaginal lumen, the expected protective effect of immunoglobulins, complement proteins, antimicrobial peptides, peroxide production, and lactic acid. In dysbiosis, the protective effect is lost, and the inflammatory response is due to bacterial proteins, increasing cell death inflammatory mediators. This increase in inflammatory mediators leads to a decrease in vaginal tolerogenic milieu, which is the response to the reduction of annidation and increase of pregnancy loss.

Figure 1 represents a summary of the events involving immune response that occur in eubiosis, normal conditions, and dysbiosis in the vagina. The interaction of microbiota and immune cells is complex. Several well-designed trials are needed to unravel the specificity of the physiological and pathological interactions that can be targeted pharmacologically. 

## 5. Perspectives of Microbiota Modulation on RPL

The main problem in understanding the changes in local microbiota is the analysis of cultured samples. The molecular diagnosis assessment should aid in defining quantitative differences in the species encountered. In general, the changes in specific species of *Lactobacillus* and the increase in *L. crispatus* in women with successful pregnancy means that local microbiota changes may be necessary but not essential only if the immune response is able to resolve the infection [4,8,11,132,133]. It is also difficult to ascertain the possible consequences of hormones like progesterone [36] and the relationship between different microbiotas, oral, gut, vaginal, and endometrial, based on current knowledge [4,6,8,132,133]. The role of sperm microbiota in RPL is only partially understood. Sperm motility seems to be affected by *Lactobacillus iners* and sperm concentration by *Pseudomonas stutzeri* and *Pseudomonas fluorescens* [133]. Thus, guidelines are required for appropriate sample collection, interpretation, and data analysis to reach a consensus and facilitate possible treatment guidelines for some RPL patients. 

Recent evidence indicates that diverse populations with a high proportion of *Lactobacillus crispatus* are positively associated with fewer infections, implantation failures (RIF), and RPL [6,57,134]. Efforts to modulate and improve the bacterial population in the vagina through the administration of antibiotics, boric acid, lactic acid, and estrogen have been unsuccessful long-term [135]. Sex hormones may alter vaginal colonization, as shown in the mouse model [136], and using a low dose of estrogen in women with atrophic vaginitis [137]. Metronidazole treatment may also affect the process [138]. Vaginal microbiota transplants are a relatively new yet promising form of therapy and consist of transferring the entire vaginal microbiota of healthy women to patients [138,139,140,141]. A proof-of-concept case study where a woman successfully shifted her microbiota population after microbiota transplant from mostly *Gardnerella* spp. (90%) to 81.2% *Lactobacillus crispatus* and 9% *Lactobacillus jensenii* ameliorated her vaginal symptoms (vaginal irritation and discharge), and she was able to carry a healthy pregnancy to term after three pregnancy losses [143]. However, this topic and the therapies available are still relatively new, and there is room for improvement in the future; clear guidelines are needed. 

Supplementary probiotics may aid vaginal and endometrial microbiota, according to reports [144,145,146]. The probiotic treatment benefits couples with RPL because of its capacity to improve aberrant spermatozoa antigenicity [146]. Tersigni and coworkers [99] reported that patients with celiac disease might benefit from oral probiotics by decreasing intestinal inflammation and increasing anti-inflammatory metabolites, reducing peripheral inflammation. Oral probiotic supplementation seems to aid vaginal microbiota [145,146,147,148,149,150]. There are still controversies in the field [70,149]. The number of well-designed clinical trials must be increased to ascertain the benefits of oral microbiota supplementation. 

The connection between imbalances in gut bacteria and inflammatory conditions mediated by Th1/Th17 in RPL has been proven [62,150]. Li Z. et al. [150] suggest that metabolites from gut microbiota impact circulating lymphocytes and may influence the migration of inflammatory cells to the endometria, altering the tolerogenic milieu formed by uterine NK cells, dendritic cells, macrophages [113,151], and T-reg cells. [152]. In RPL patients, this tolerogenic complex is reduced [114,150,151,152]. The gut bacteria of these patients have been shown to produce lower levels of deoxycholic acid (DCA), glycolithocholic acid (GLCA), acetate, propionate, and butyrate [153,154]. These findings suggest a connection between the bile acids and short-chain fatty acids levels produced by gut bacteria with the circulating T and B cell subpopulations. 

Analysis of the fecal microbiome revealed lower microbial diversity and decreased levels of *Prevotella*_1, *Prevotellaceae*_UCG_003, and *Selenomonas*_1 in these patients [150]. Correlation analyses showed that specific microbe-related metabolites were positively linked to changes in Th1/Th17 cytokine levels in the miscarriage group [150,155]. Additionally, imidazolepropionic acid and 1,4-methylimidazoleacetic acid were identified as being associated with subsequent recurrent miscarriage. [150]. The reduction in butyrate-producing bacteria in the gut microbiota of RPL demonstrated a link between immune vigilant responses and anti-inflammatory properties of the microbiota [150,153,154,155]. A report studied 20 fecal samples of patients with RPL, and antibody-related factors explained the conditions compared to 20 patients without antibodies [156]. Community richness and phylogenetic diversity in the antibody-positive group were higher than in the opposing group. The *Bacteroides* genera were prevalent in the positive group. In contrast, in the opposing group, *Bacteroides* was less prevalent, and bacteria of the genera *Erysipelotrichaceae*, *Faecalibacterium*, *Enterococcus*, *Prevotella*, *Megasphaera*, and *Anaerostipes* were also encountered [157]. Proteomic studies support the results and propose that cytoskeleton proteins may be crucial in the local disarrangements in endometrial tissue, which can lead to a lack of implantation [157]. In summary, well-defined clinical trials are required to identify the importance of gut microbiota and its possible therapeutic role in RPL. 

The serum of miscarriage patients has been shown to contain significantly higher levels of specific inflammatory cytokines (IL-2, IL-17A, IL-17F, TNF-α, and IFN-γ) [158,159]. Probiotics activate anti-inflammatory mechanisms by producing anti-inflammatory cytokines such as IL-4, IL-10, IL-11, and IL-13 while hindering pro-inflammatory cytokines such as IL-1, IL-6, and TNF-α [159,160]. This increases circulating regulatory Tregs and Th2 cells, decreasing the pro-inflammatory Th1 and Th17 subpopulations. Various strains of probiotics, including *Lactobacillus plantarum*, *Lactobacillus rhamnosus*, *Lactobacillus casei*, *Lactobacillus reuteri*, *Bifidobacterium longum*, and *Bifidobacterium breve*, have been identified as potential treatments for several medical conditions [159,160,161,162]. Probiotics stabilize the gut’s physiological responses, stabilizing the interaction of enterocytes, preventing the permeation of bacterial toxins (leaky syndrome), and not stimulating the local immune cells, creating a tolerogenic environment associated with a decreased peripheral inflammatory response. Engineered probiotics have been designed to treat different chronic conditions [163], and clinical trials should provide critical information about their benefits.

Prednisone treatment reduces the local inflammatory response, consequently decreasing abortion risk and increasing pregnancy efficiency in IVF [164,165], along with the use of nutritional supplements [166]. Also, anti-inflammatory cytokines [167], topic cytokines such as G-CSF [168], and interferon λ [169] can increase the efficiency of implantation and, consequently, pregnancy based on the induction of tolerance at the tissue level. However, critical scientific evidence of therapeutic local progesterone and cytokines with local microbiota has not been documented.

Omega-3 supplementation has been shown to increase fertility in mice [170] and is now being successfully used to enhance human fertility [171] and possibly reduce RLP [172]. However, in their review, Kello and Cho [173] mentioned that supplementation may only benefit patients with antiphospholipid syndrome. Consequently, it may assist a group of patients with RPL in which immune disorders may be involved. How oral supplementation of omega-3 may affect local microbiota is unknown; however, it can be postulated that a decrease in prostaglandin E2 production, as a product of an inflammatory response, may promote/support the production of other anti-inflammatory intermediates. The role of nutritional supplementation on RPL should be carefully studied [4,6,147,162,166]. Chen P et al. [174] concluded that harmful local bacteria could produce vast amounts of eicosapentaenoic acid (EPA), which may be responsible for a decrease in zygote implantation. However, the primary analysis used bioinformatics instead of lipidomics, generating doubts about the validity of the conclusions. 

Izadifar Z and coworkers [175] recently reported a cervix chip for studying the physiological responses of bacteria and immune cells and interactions with endometrial tissue. Colonizing the Cervix Chip with *L. crispatus* bacteria increased mucus layer thickness and quality compared to *G vaginalis*. The effect parallels the results recorded in the clinic. The chip can help assess the role of immune cells, antibodies, and anti-microbicidal peptides in the absence and presence of infection. Since mucus conditions change upon infection, the local barrier and innate response could be impaired, generating the chemoattraction of polymorphonuclear cells and other lymphocytes at the site of the inflammatory response. In addition, the chip may allow the analysis of the effect of estrogen and progesterone in the local infection and the modulation of the immune response. Thus, the chip facilitates the study of the microbiota in the local tissue and may provide new elements for understanding the role of immune cells in the process and how pharmacological therapies may aid in generating a suitable milieu for zygote anidation and growth. 

## 6. Conclusions

Primary and secondary RPL are complex medical conditions involving 1–2% of women of reproductive age. Despite numerous efforts to study the possible triggering factors, significant achievements have yet to be reported. Local (vaginal and endometrial) and gut microbiota and metabolites differ in RPL compared to normal pregnancies. Specific *Lactobacillus species*, *L. crispatus*, and *L. jensenii*, are critical to protecting the host from local infection and possible leaky syndrome.

There is evidence of immune cell migration and local tissue disarrangements in RPL as a response to the inflammatory conditions generated either by vaginal or endometrial dysbiosis. It cannot be ruled out that the genetic conditions of the host and local infections are responsible for molecular mimicry and autoimmunity, which can be involved in the pathogenesis of RPL. Protective microbiota prevents local inflammatory response and infections and protects endometrial tissue. Transplantation of microbiota and oral probiotics may help maintain vaginal microbiota. Patients with RPL, especially those with immune disorders, may benefit from oral probiotic supplementation or microbiota transplantation. It is not clear whether antigen mimicry can be prevented by treatment. Also, the modulation of gut microbiota may be useful in preventing other medical conditions that affect RIF and RPL. 

The analysis of endometrial microbiota in clinical assessments can optimize treatment strategies in assisted reproductive technology protocols. Microbiota profiles can personalize therapeutic approaches and improve treatment outcomes for patients facing reproductive challenges. It is possible that immune modulation through microbiota can enhance the tolerogenic immune response required for a normal pregnancy. Implementing microbiota analysis in clinical practice may present challenges, such as the need for standardized guidelines and addressing logistical considerations; however, it may represent a significant opportunity to increase implantation and successful full-term pregnancies. 

Well-planned clinical trials are required to provide evidence of the role of microbiota and microbiota transplantation in RPL. RPL patients may be at risk of developing medical conditions shortly after reproductive age; therefore, studies on RPL classification and triggers are urgently required.

## Figures and Tables

**Table 1:** Summary of the reports referring vaginal or endometrial microbiota with miscarriage or RPL.

Findings	References
Low levels of *Lactobacillus* spp. in the first trimester of pregnancy may be associated with a higher risk of pregnancy loss in the second trimester.	[43]
The risk of miscarriage increases when vaginal *Lactobacillus* spp. levels fall during the first or second trimester of pregnancy.	[44,45]
Women with abnormal vaginal microbiota, i.e., *Gardnerella vaginalis* and *Ureaplasma* spp. have a higher risk of preterm birth and probably miscarriage.	[46]
A higher abundance of pathogenic bacteria, such as the genera *Ureaplasma* and *Mycoplasma*, was found in women who had a miscarriage.	[47]
Vaginal dysbiosis frequency was higher in women who had experienced a second-trimester miscarriage compared to those who had multiple miscarriages.	[48]
Women who experienced one miscarriage in the previous six months had been suffering from bacterial vaginosis.	[49]
*Lactobacillus* spp. is absent in vaginal samples from RPL patients.	[50]
Women who had a miscarriage had higher levels of potentially pathogenic bacteria in their vaginal microbiota compared to women with successful pregnancies.	[51]
Reduced *Lactobacillus* spp. levels are associated with the growth of bacteria of the genera *Streptococcus*, *Prevotella*, and *Atopobium* in women with RPL.	[52]
The decreased amount of *Lactobacillus* spp. and an increased number of bacteria of the genera *Gardnerella*, *Prevotella*, *Megastrobila*, and *Cyclospora* in vaginal microbiota may be responsible for RPL.	[53]
*Ureaplasma* spp. is abundant in the endometrial microbiota of RPL patients	[54]
The presence of *Lactobacillus iners* in vaginal microbiota increases the possibility of miscarriage.	[55]
High prevalence of antibiotic-resistant *Enterococcus* spp. and *Staphylococcus* spp. in the vaginal flora RPL patients	[56]
Low quantity of *Lactobacillus* species in samples of uterine microbiota in RIF and RPL	[57]
A dysbiotic endometrial microbiota profile composed of the genera *Atopobium*, *Bifidobacterium*, *Chryseobacterium*, *Gardnerella*, *Haemophilus*, *Klebsiella*, *Neisseria*, *Staphylococcus* and *Streptococcus* was associated with miscarriage.	[58]
Lower levels of *Lactobacillus crispatus* and high levels of pathogenic bacteria in patients with RPL as compared to normal pregnancy	[59]
*Gardnerella vaginalis* was present in higher abundance in the endometrial samples of women with RPL than in the controls.	[60]
*Pseudomonadota* and *Bacillota* species were elevated in the endometrial microbiota of RPL patients.	[61]
Infertile women with chronic endometritis have reduced amounts of *Bifidobacterium* and lactic acid-producing bacteria in their vaginal microbiota, apart from *Lactobacillus*.	[62]
Patients with RPL had lower levels of *Lactobacillus* spp. and abundant levels of pathogenic bacteria in the cervical mucus than women with successful pregnancies.	[63]
Bacteria of the genera *Cutibacterium* and *Anaerobacillus* are abundant in the cervix of patients with miscarriage compared to normal pregnancy.	[64]
*Lactobacillus acidophilus* was absent, but *Lactobacillus iners* was abundant in the vaginal and endometrial samples of RPL women with celiac disease.	[65]
*L. jensenii* was decreased in the early embryonic arrest group compared to the normal pregnancy cohort	[66]
Vaginal dysbiosis correlates with a higher pregnancy loss in IVF patients	[67]

## Data Availability

Not applicable.

## References

[1] Stephenson M.D. (1996). Frequency of factors associated with habitual abortion in 197 couples. Fertil. Steril..

[2] Ford H.B., Schust D.J. (2009). Recurrent pregnancy loss: Etiology, diagnosis, and therapy. Rev. Obstet. Gynecol..

[3] Pillarisetty L.S., Mahdy H. (2024). Recurrent Pregnancy Loss. [Updated 2023 August 28]. StatPearls.

[4] Gao H., Liu Q., Wang X., Li T., Li H., Li G., Tan L., Chen Y. (2024). Deciphering the role of female reproductive tract microbiome in reproductive health: A review. Front. Cell. Infect. Microbiol..

[5] Agostinis C., Mangogna A., Bossi F., Ricci G., Kishore U., Bulla R. (2019). Uterine Immunity and Microbiota: A Shifting Paradigm. Front. Immunol..

[6] Gao X., Louwers Y.V., Laven J.S.E., Schoenmakers S. (2024). Clinical Relevance of Vaginal and Endometrial Microbiome Investigation in Women with Repeated Implantation Failure and Recurrent Pregnancy Loss. Int. J. Mol. Sci..

[7] Lee S.K., Kim C.J., Kim D.J., Kang J.H. (2015). Immune cells in the female reproductive tract. Immune Netw..

[8] Mendz G.L. (2023). The Vaginal Microbiome during Pregnancy in Health and Disease. Appl. Microbiol..

[9] Benner M., Ferwerda G., Joosten I., van der Molen R.G. (2018). How uterine microbiota might be responsible for a receptive, fertile endometrium. Hum. Reprod. Update.

[10] Al-Nasiry S., Ambrosino E., Schlaepfer M., Morré S.A., Wieten L., Voncken J.W., Spinelli M., Mueller M., Kramer B.W. (2020). The interplay between reproductive tract microbiota and immunological system in human reproduction. Front. Immunol..

[11] Odendaal J., Black N., Bennett P.R., Brosens J., Quenby S., MacIntyre D.A. (2024). The endometrial microbiota and early pregnancy loss. Hum. Reprod..

[12] Lev-Sagie A., De Seta F., Verstraelen H., Ventolini G., Lonnee-Hoffmann R., Vieira-Baptista P. (2022). The Vaginal Microbiome: II. Vaginal Dysbiotic Conditions. J. Low. Genit. Tract Dis..

[13] Elnaggar J.H., Ardizzone C.M., Cerca N., Toh E., Łaniewski P., Lillis R.A., Herbst-Kralovetz M.M., Quayle A.J., Muzny C.A., Taylor C.M. (2023). A novel Gardnerella, Prevotella, and Lactobacillus standard that improves accuracy in quantifying bacterial burden in vaginal microbial communities. Front. Cell. Infect. Microbiol..

[14] Sola-Leyva A., Andrés-León E., Molina N.M., Terron-Camero L.C., Plaza-Díaz J., Sáez-Lara M.J., Gonzalvo M.C., Sánchez R., Ruíz S., Martínez L. (2021). Mapping the entire functionally active endometrial microbiota. Hum. Reprod..

[15] Blazheva S., Pachkova S., Bodurska T., Ivanov P., Blazhev A., Lukanov T., Konova E. (2024). Unlocking the Uterine Code: Microbiota, Immune Cells, and Therapy for Recurrent Reproductive Failure. Microorganisms.

[16] Brown R.G., Al-Memar M., Marchesi J.R., Lee Y.S., Smith A., Chan D., Lewis H., Kindinger L., Terzidou V., Bourne T. (2019). Establishment of vaginal microbiota composition in early pregnancy and its association with subsequent preterm prelabor rupture of the fetal membranes. Transl. Res..

[17] Song S.D., Acharya K.D., Zhu J.E., Deveney C.M., Walther-Antonio M.R.S., Tetel M.J., Chia N. (2020). Daily Vaginal Microbiota Fluctuations Associated with Natural Hormonal Cycle, Contraceptives, Diet, and Exercise. mSphere.

[18] Toson B., Simon C., Moreno I. (2022). The Endometrial Microbiome and Its Impact on Human Conception. Int. J. Mol. Sci..

[19] Lewis F.M.T., Bernstein K.T., Aral S.O. (2017). Vaginal Microbiome and Its Relationship to Behavior, Sexual Health, and Sexually Transmitted Diseases. Obstet. Gynecol..

[20] Conlon M.A., Bird A.R. (2014). The impact of diet and lifestyle on gut microbiota and human health. Nutrients.

[21] Holdcroft A.M., Ireland D.J., Payne M.S. (2023). The Vaginal Microbiome in Health and Disease—What Role Do Common Intimate Hygiene Practices Play?. Microorganisms.

[22] Ma Z.S. (2022). Microbiome Transmission During Sexual Intercourse Appears Stochastic and Supports the Red Queen Hypothesis. Front. Microbiol..

[23] McClelland R.S., Lingappa J.R., Srinivasan S., Kinuthia J., John-Stewart G.C., Jaoko W., Richardson B.A., Yuhas K., Fiedler T.L., Mandaliya K.N. (2018). Evaluation of the association between the concentrations of key vaginal bacteria and the increased risk of HIV acquisition in African women from five cohorts: A nested case-control study. Lancet Infect. Dis..

[24] Chacra L.A., Ly C., Hammoud A., Iwaza R., Mediannikov O., Bretelle F., Fenollar F. (2023). Relationship between Bacterial Vaginosis and Sexually Transmitted Infections: Coincidence, Consequence or Co-Transmission?. Microorganisms.

[25] Wang Y., Thakur R., Shen Q., He Y., Chen C. (2023). Influences of vaginal microbiota on human papillomavirus infection and host immune regulation: What we have learned?. Decod. Infect. Transm..

[26] Zeng M., Li X., Jiao X., Cai X., Yao F., Xu S., Huang X., Zhang Q., Chen J. (2023). Roles of vaginal flora in human papillomavirus infection, virus persistence and clearance. Front. Cell. Infect. Microbiol..

[27] Lebeau A., Bruyere D., Roncarati P., Peixoto P., Hervouet E., Cobraiville G., Taminiau B., Masson M., Gallego C., Mazzucchelli G. (2022). HPV infection alters vaginal microbiome through down-regulating host mucosal innate peptides used by Lactobacilli as amino acid sources. Nat. Commun..

[28] Mehta S.D., Nandi D., Agingu W., Green S.J., Bhaumik D.K., Bailey R.C., Otieno F. (2022). Vaginal and Penile Microbiome Associations With Herpes Simplex Virus Type 2 in Women and Their Male Sex Partners. J. Infect. Dis..

[29] Brotman R.M., Klebanoff M.A., Nansel T.R., Yu K.F., Andrews W.W., Zhang J., Schwebke J.R. (2010). Bacterial vaginosis assessed by gram stain and diminished colonization resistance to incident gonococcal, chlamydial, and trichomonal genital infection. J. Infect. Dis..

[30] Van Gerwen O.T., Muzny C.A., Marrazzo J.M. (2022). Sexually transmitted infections and female reproductive health. Nat. Microbiol..

[31] Haggerty C.L., Ness R.B., Totten P.A., Farooq F., Tang G., Ko D.B., Hou X., Fiedler T.L.B., Srinivasan S., Astete S.G. (2020). Presence and Concentrations of Select Bacterial Vaginosis-Associated Bacteria Are Associated With Increased Risk of Pelvic Inflammatory Disease. Sex. Transm. Dis..

[32] Brown S.E., Schwartz J.A., Robinson C.K., O’Hanlon D.E., Bradford L.L., He X., Mark K.S., Bruno V.M., Ravel J., Brotman R.M. (2019). The Vaginal Microbiota and Behavioral Factors Associated With Genital Candida albicans Detection in Reproductive-Age Women. Sex. Transm. Dis..

[33] Sobstyl A., Chałupnik A., Mertowska P., Grywalska E. (2023). How Do Microorganisms Influence the Development of Endometriosis? Participation of Genital, Intestinal and Oral Microbiota in Metabolic Regulation and Immunopathogenesis of Endometriosis. Int. J. Mol. Sci..

[34] Gao Q., Fan T., Luo S., Zheng J., Zhang L., Cao L., Zhang Z., Li L., Huang Z., Zhang H. (2023). Lactobacillus gasseri LGV03 isolated from the cervico-vagina of HPV-cleared women modulates epithelial innate immune responses and suppresses the growth of HPV-positive human cervical cancer cells. Transl. Oncol..

[35] Krog M.C., Hugerth L.W., Fransson E., Bashir Z., Andersen A.N., Edfeldt G., Engstrand L., Schuppe-Koistinen I., Nielsen H.S. (2022). The healthy female microbiome across body sites: Effect of hormonal contraceptives and the menstrual cycle. Hum. Reprod..

[36] van den Tweel M.M., van den Munckhof E.H.A., van der Zanden M., Molijn A.C., van Lith J.M.M., Le Cessie S., Boers K.E. (2024). Bacterial vaginosis in a subfertile population undergoing fertility treatments: A prospective cohort study. J. Assist. Reprod. Genet..

[37] Wang T., Li P., Bai X., Tian S., Yang M., Leng D., Kui H., Zhang S., Yan X., Zheng Q. (2024). Vaginal microbiota are associated with in vitro fertilization during female infertility. iMeta.

[38] Elnashar A.M. (2021). Impact of endometrial microbiome on fertility. Middle East Fertil. Soc. J..

[39] Hugon A.M., Golos T.G. (2023). Non-human primate models for understanding the impact of the microbiome on pregnancy and the female reproductive tract†. Biol. Reprod..

[40] Schuster H.J., Bos A.M., Himschoot L., van Eekelen R., Matamoros S.P., de Boer M.A., Oudijk M.A., Ris-Stalpers C., Cools P., Savelkoul P.H. (2024). Vaginal microbiota and spontaneous preterm birth in pregnant women at high risk of recurrence. Heliyon.

[41] Sun S., Serrano M.G., Fettweis J.M., Basta P., Rosen E., Ludwig K., Sorgen A.A., Blakley I.C., Wu M.C., Dole N. (2022). Race, the Vaginal Microbiome, and Spontaneous Preterm Birth. mSystems.

[42] Saadaoui M., Singh P., Ortashi O., Al Khodor S. (2023). Role of the vaginal microbiome in miscarriage: Exploring the relationship. Front. Cell. Infect. Microbiol..

[43] Nelson D.B., Bellamy S., Nachamkin I., Ness R.B., Macones G.A., Allen-Taylor L. (2007). First trimester bacterial vaginosis, individual microorganism levels, and risk of second trimester pregnancy loss among urban women. Fertil. Steril..

[44] Nelson D.B., Hanlon A., Nachamkin I., Haggerty C., Mastrogiannis D.S., Liu C., Fredricks D.N. (2014). Early Pregnancy Changes in Bacterial Vaginosis-Associated Bacteria and Preterm Delivery. Paediatr. Perinat. Epidemiology.

[45] Nelson D.B., Hanlon A.L., Wu G., Liu C., Fredricks D.N. (2015). First Trimester Levels of BV-Associated Bacteria and Risk of Miscarriage Among Women Early in Pregnancy. Matern. Child Health J..

[46] DiGiulio D.B., Callahan B.J., McMurdie P.J., Costello E.K., Lyell D.J., Robaczewska A., Sun C.L., Goltsman D.S.A., Wong R.J., Shaw G. (2015). Temporal and spatial variation of the human microbiota during pregnancy. Proc. Natl. Acad. Sci. USA.

[47] Ahmadi A., Khodabandehloo M., Ramazanzadeh R., Farhadifar F., Nikkhoo B., Soofizade N., Rezaii M. (2014). Association between Ureaplasma urealyticum endocervical infection and spontaneous abortion. Iran. J. Microbiol..

[48] McPherson E. (2016). Recurrence of stillbirth and second trimester pregnancy loss. Am. J. Med Genet. A.

[49] Işik G., Demirezen Ş., Dönmez H.G., Beksaç M.S. (2016). Bacterial vaginosis in association with spontaneous abortion and recurrent pregnancy losses. J. Cytol..

[50] Kuon R.J., Togawa R., Vomstein K., Weber M., Goeggl T., Strowitzki T., Markert U.R., Zimmermann S., Daniel V., Dalpke A.H. (2017). Higher prevalence of colonization with *Gardnerella vaginalis* and gram-negative anaerobes in patients with recurrent miscarriage and elevated peripheral natural killer cells. J. Reprod. Immunol..

[51] Al-Memar M., Bobdiwala S., Fourie H., Mannino R., Lee Y., Smith A., Marchesi J., Timmerman D., Bourne T., Bennett P. (2020). The association between vaginal bacterial composition and miscarriage: A nested case-control study. BJOG.

[52] Chang D.H., Shin J., Rhee M.S., Park K.R., Cho B.K., Lee S.K., Kim B.C. (2020). Vaginal Microbiota Profiles of Native Korean Women and Associations with High-Risk Pregnancy. J. Microbiol. Biotechnol..

[53] Xu L., Huang L., Lian C., Xue H., Lu Y., Chen X., Xia Y. (2020). Vaginal Microbiota Diversity of Patients with Embryonic Miscarriage by Using 16S rDNA High-Throughput Sequencing. Int. J. Genom..

[54] Shi Y., Yamada H., Sasagawa Y., Tanimura K., Deguchi M. (2022). Uterine endometrium microbiota and pregnancy outcome in women with recurrent pregnancy loss. J. Reprod. Immunol..

[55] Shahid M., Quinlivan J.A., Peek M., Castaño-Rodríguez N., Mendz G.L. (2022). Is there an association between the vaginal microbiome and first-trimester miscarriage? A prospective observational study. J. Obstet. Gynaecol. Res..

[56] Ncib K., Bahia W., Leban N., Mahdhi A., Trifa F., Mzoughi R., Haddad A., Jabeur C., Donders G. (2022). Microbial Diversity and Pathogenic Properties of Microbiota Associated with Aerobic Vaginitis in Women with Recurrent Pregnancy Loss. Diagnostics.

[57] Vomstein K., Reider S., Böttcher B., Watschinger C., Kyvelidou C., Tilg H., Moschen A.R., Toth B. (2022). Uterine microbiota plasticity during the menstrual cycle: Differences between healthy controls and patients with recurrent miscarriage or implantation failure. J. Reprod. Immunol..

[58] Moreno I., Garcia-Grau I., Perez-Villaroya D., Gonzalez-Monfort M., Bahçeci M., Barrionuevo M.J., Taguchi S., Puente E., Dimattina M., Lim M.W. (2022). Endometrial microbiota composition is associated with reproductive outcome in infertile patients. Microbiome.

[59] Severgnini M., Morselli S., Camboni T., Ceccarani C., Laghi L., Zagonari S., Patuelli G., Pedna M.F., Sambri V., Foschi C. (2022). A Deep Look at the Vaginal Environment During Pregnancy and Puerperium. Front. Cell. Infect. Microbiol..

[60] Peuranpää P., Holster T., Saqib S., Kalliala I., Tiitinen A., Salonen A., Hautamäki H. (2022). Female reproductive tract microbiota and recurrent pregnancy loss: A nested case-control study. Reprod. Biomed. Online.

[61] Shu J., Lin S., Wu Y., Zhu J., Gong D., Zou X., Zhu H., Gao J. (2022). A potential role for the uterine microbiome in missed abortions. J. Biol. Regul. Homeost. Agents.

[62] Tanaka S.E., Sakuraba Y., Kitaya K., Ishikawa T. (2022). Differential Vaginal Microbiota Profiling in Lactic-Acid-Producing Bacteria between Infertile Women with and without Chronic Endometritis. Diagnostics.

[63] Dong M., Dong Y., Bai J., Li H., Ma X., Li B., Wang C., Li H., Qi W., Wang Y. (2023). Interactions between microbiota and cervical epithelial, immune, and mucus barrier. Front. Cell. Infect. Microbiol..

[64] Mori R., Hayakawa T., Hirayama M., Ozawa F., Yoshihara H., Goto S., Kitaori T., Ozaki Y., Sugiura-Ogasawara M. (2023). Cervicovaginal microbiome in patients with recurrent pregnancy loss. J. Reprod. Immunol..

[65] Masucci L., D’Ippolito S., De Maio F., Quaranta G., Mazzarella R., Bianco D.M., Castellani R., Inversetti A., Sanguinetti M., Gasbarrini A. (2023). Celiac Disease Predisposition and Genital Tract Microbiota in Women Affected by Recurrent Pregnancy Loss. Nutrients.

[66] Wang Y., Wang X., Zhu M., Ge L., Liu X., Su K., Chen Z., Zhao W. (2022). The Interplay Between Cervicovaginal Microbial Dysbiosis and Cervicovaginal Immunity. Front. Immunol..

[67] Celicanin M.M., Haahr T., Humaidan P., Skafte-Holm A. (2024). Vaginal dysbiosis—The association with reproductive outcomes in IVF patients: A systematic review and meta-analysis. Curr. Opin. Obstet. Gynecol..

[68] Grewal K., Lee Y.S., Smith A., Brosens J.J., Bourne T., Al-Memar M., Kundu S., MacIntyre D.A., Bennett P.R. (2022). Chromosomally normal miscarriage is associated with vaginal dysbiosis and local inflammation. BMC Med..

[69] Liu Y., Chen H., Feng L., Zhang J. (2021). Interactions between gut microbiota and metabolites modulate cytokine network imbalances in women with unexplained miscarriage. NPJ Biofilms Microbiomes.

[70] Vomstein K., Krog M.C., Wrønding T., Nielsen H.S. (2024). The microbiome in recurrent pregnancy loss—A scoping review. J. Reprod. Immunol..

[71] Liu F.T., Yang S., Yang Z., Zhou P., Peng T., Yin J., Ye Z., Shan H., Yu Y., Li R. (2022). An Altered Microbiota in the Lower and Upper Female Reproductive Tract of Women with Recurrent Spontaneous Abortion. Microbiol. Spectr..

[72] Wang L., Chen J., He L., Liu H., Liu Y., Luan Z., Li H., Liu W., Luo M. (2023). Association between the vaginal and uterine microbiota and the risk of early embryonic arrest. Front. Microbiol..

[73] Takimoto K., Yamada H., Shimada S., Fukushi Y., Wada S. (2023). Chronic Endometritis and Uterine Endometrium Microbiota in Recurrent Implantation Failure and Recurrent Pregnancy Loss. Biomedicines.

[74] Palomino M.M., Allievi M.C., Gordillo T.B., Bockor S.S., Fina Martin J., Ruzal S.M. (2023). Surface layer proteins in species of the family Lactobacillaceae. Microb. Biotechnol..

[75] France M., Alizadeh M., Brown S., Ma B., Ravel J. (2022). Towards a deeper understanding of the vaginal microbiota. Nat. Microbiol..

[76] Mendes-Soares H., Suzuki H., Hickey R.J., Forney L.J. (2014). Comparative functional genomics of Lactobacillus spp. reveals possible mechanisms for specialization of vaginal lactobacilli to their environment. J. Bacteriol..

[77] Smith S.B., Ravel J. (2017). The vaginal microbiota, host defence and reproductive physiology. J. Physiol..

[78] Zheng N., Guo R., Wang J., Zhou W., Ling Z. (2021). Contribution of Lactobacillus iners to Vaginal Health and Diseases: A Systematic Review. Front. Cell. Infect. Microbiol..

[79] Cela V., Daniele S., Obino M.E.R., Ruggiero M., Zappelli E., Ceccarelli L., Papini F., Marzi I., Scarfò G., Tosi F. (2022). Endometrial Dysbiosis Is Related to Inflammatory Factors in Women with Repeated Implantation Failure: A Pilot Study. J. Clin. Med..

[80] Santoro A., Travaglino A., Inzani F., Angelico G., Raffone A., Maruotti G.M., Straccia P., Arciuolo D., Castri F., D’Alessandris N. (2023). The Role of Plasma Cells as a Marker of Chronic Endometritis: A Systematic Review and Meta-Analysis. Biomedicines.

[81] Ma N., Li J., Zhang J., Jin Y., Wang J., Qin W., Hang F., Qin A. (2023). Combined oral antibiotics and intrauterine perfusion can improve in vitro fertilization and embryo transfer pregnancy outcomes in patients with chronic endometritis and repeated embryo implantation failure. BMC Women’s Health.

[82] Kitaya K., Yasuo T. (2023). Commonalities and Disparities between Endometriosis and Chronic Endometritis: Therapeutic Potential of Novel Antibiotic Treatment Strategy against Ectopic Endometrium. Int. J. Mol. Sci..

[83] Christiansen O.B., Steffensen R., Nielsen H.S., Varming K. (2008). Multifactorial etiology of recurrent miscarriage and its scientific and clinical implications. Gynecol. Obstet. Investig..

[84] Ishimwe J.A. (2021). Maternal microbiome in preeclampsia pathophysiology and implications on offspring health. Physiol. Rep..

[85] Rafat D., Singh S., Nawab T., Khan F., Khan A.U., Khalid S. (2022). Association of vaginal dysbiosis and gestational diabetes mellitus with adverse perinatal outcomes. Int. J. Gynecol. Obstet..

[86] Kan H., He Y., Li Q., Mu Y., Dong Y., Fan W., Zhang M., Wang T., Li Y., Liu H. (2022). Differential Effect of Vaginal Microbiota on Spontaneous Preterm Birth among Chinese Pregnant Women. BioMed Res. Int..

[87] Esmaeili S.A., Mahmoudi M., Rezaieyazdi Z., Sahebari M., Tabasi N., Sahebkar A., Rastin M. (2018). Generation of tolerogenic dendritic cells using Lactobacillus rhamnosus and Lactobacillus delbrueckii as tolerogenic probiotics. J. Cell. Biochem..

[88] Qi X., Yun C., Pang Y., Qiao J. (2021). The impact of the gut microbiota on the reproductive and metabolic endocrine system. Gut Microbes.

[89] Sun Y., Gao S., Ye C., Zhao W. (2023). Gut microbiota dysbiosis in polycystic ovary syndrome: Mechanisms of progression and clinical applications. Front. Cell. Infect. Microbiol..

[90] Corrie L., Awasthi A., Kaur J., Vishwas S., Gulati M., Kaur I.P., Gupta G., Kommineni N., Dua K., Singh S.K. (2023). Interplay of Gut Microbiota in Polycystic Ovarian Syndrome: Role of Gut Microbiota, Mechanistic Pathways and Potential Treatment Strategies. Pharmaceuticals.

[91] Fettweis J.M., Serrano M.G., Brooks J.P., Edwards D.J., Girerd P.H., Parikh H.I., Huang B., Arodz T.J., Edupuganti L., Glascock A.L. (2019). The vaginal microbiome and preterm birth. Nat. Med..

[92] Zhu J., Jin J., Qi Q., Li L., Zhou J., Cao L., Wang L. (2023). The association of gut microbiome with recurrent pregnancy loss: A comprehensive review. Drug Discov. Ther..

[93] Soyer Caliskan C., Yurtcu N., Celik S., Sezer O., Kilic S.S., Cetin A. (2022). Derangements of vaginal and cervical canal microbiota determined with real-time PCR in women with recurrent miscarriages. J. Obstet. Gynaecol..

[94] Song D., He Y., Wang Y., Liu Z., Xia E., Huang X., Xiao Y., Li T.-C. (2021). Impact of antibiotic therapy on the rate of negative test results for chronic endometritis: A prospective randomized control trial. Fertil. Steril..

[95] Salmeri N., Sinagra E., Dolci C., Buzzaccarini G., Sozzi G., Sutera M., Candiani M., Ungaro F., Massimino L., Danese S. (2023). Microbiota in Irritable Bowel Syndrome and Endometriosis: Birds of a Feather Flock Together—A Review. Microorganisms.

[96] Peelen M.J., Luef B.M., Lamont R.F., de Milliano I., Jensen J.S., Limpens J., Hajenius P.J., Jørgensen J.S., Menon R., PREBIC Biomarker Working Group 2014–2018 (2019). The influence of the vaginal microbiota on preterm birth: A systematic review and recommendations for a minimum dataset for future research. Placenta.

[97] Ghosh S.S., Wang J., Yannie P.J., Ghosh S. (2020). Intestinal Barrier Dysfunction, LPS Translocation, and Disease Development. J. Endocr. Soc..

[98] den Besten G., van Eunen K., Groen A.K., Venema K., Reijngoud D.-J., Bakker B.M. (2013). The role of short-chain fatty acids in the interplay between diet, gut microbiota, and host energy metabolism. J. Lipid Res..

[99] Tersigni C., D’Ippolito S., Di Nicuolo F., Marana R., Valenza V., Masciullo V., Scaldaferri F., Malatacca F., de Waure C., Gasbarrini A. (2018). Recurrent pregnancy loss is associated to leaky gut: A novel pathogenic model of endometrium inflammation?. J. Transl. Med..

[100] Charoensappakit A., Sae-Khow K., Leelahavanichkul A. (2022). Gut Barrier Damage and Gut Translocation of Pathogen Molecules in Lupus, an Impact of Innate Immunity (Macrophages and Neutrophils) in Autoimmune Disease. Int. J. Mol. Sci..

[101] Poggi A., Benelli R., Venè R., Costa D., Ferrari N., Tosetti F., Zocchi M.R. (2019). Human Gut-Associated Natural Killer Cells in Health and Disease. Front. Immunol..

[102] Pelzer E.S., Willner D., Buttini M., Huygens F. (2018). A role for the endometrial microbiome in dysfunctional menstrual bleeding. Antonie Leeuwenhoek.

[103] Tersigni C., Barbaro G., Castellani R., Onori M., Granieri C., Scambia G., Di Simone N. (2024). Oral administration of Bifidobacterium longum ES1 reduces endometrial inflammation in women with recurrent pregnancy loss. Am. J. Reprod. Immunol..

[104] Huang L., Thonusin C., Chattipakorn N., Chattipakorn S.C. (2021). Impacts of gut microbiota on gestational diabetes mellitus: A comprehensive review. Eur. J. Nutr..

[105] Belizário J.E., Faintuch J., Garay-Malpartida M. (2018). Gut Microbiome Dysbiosis and Immunometabolism: New Frontiers for Treatment of Metabolic Diseases. Mediat. Inflamm..

[106] Larsen J.M. (2017). The immune response to Prevotella bacteria in chronic inflammatory disease. Immunology.

[107] Russell J.T., Roesch L.F.W., Ördberg M., Ilonen J., Atkinson M.A., Schatz D.A., Triplett E.W., Ludvigsson J. (2019). Genetic risk for autoimmunity is associated with distinct changes in the human gut microbiome. Nat. Commun..

[108] Yang P., Lu T., Liang X., Huang T., Wu L., He Z., Xiao X., Fan S. (2024). The influence of placenta microbiota of normal term pregnant women on immune regulation during pregnancy. BMC Pregnancy Childbirth.

[109] Yang S., Wang H., Li D., Li M. (2024). An Estrogen-NK Cells Regulatory Axis in Endometriosis, Related Infertility, and Miscarriage. Int. J. Mol. Sci..

[110] López-Moreno A., Aguilera M. (2020). Probiotics Dietary Supplementation for Modulating Endocrine and Fertility Microbiota Dysbiosis. Nutrients.

[111] Murphy K., Gromisch M., Srinivasan S., Wang T., Wood L., Proll S., Liu C., Fiedler T., Valint D.J., Fredricks D.N. (2023). IgA coating of vaginal bacteria is reduced in the setting of bacterial vaginosis (BV) and preferentially targets BV-associated species. Infect. Immun..

[112] Azkargorta M., Bregón-Villahoz M., Escobes I., Ibáñez-Pérez J., Iloro I. (2020). In-depth proteomics and natural peptidomics analyses reveal antibacterial peptides in human endometrial fluid. J. Proteom..

[113] Garmendia J.V., De Sanctis J.B. (2021). A Brief Analysis of Tissue-Resident NK Cells in Pregnancy and Endometrial Diseases: The Importance of Pharmacologic Modulation. Immuno.

[114] Dai M., Xu Y., Gong G., Zhang Y. (2023). Roles of immune microenvironment in the female reproductive maintenance and regulation: Novel insights into the crosstalk of immune cells. Front. Immunol..

[115] Hedger M.P. (2015). The Immunophysiology of Male Reproduction. Knobil and Neill’s Physiology of Reproduction.

[116] Solders M., Gorchs L., Erkers T., Lundell A.C., Nava S., Gidlöf S., Tiblad E., Magalhaes I., Kaipe H. (2017). MAIT cells accumulate in placental intervillous space and display a highly cytotoxic phenotype upon bacterial stimulation. Sci. Rep..

[117] Favaro R.R., Phillips K., Delaunay-Danguy R., Ujčič K., Markert U.R. (2022). Emerging Concepts in Innate Lymphoid Cells, Memory, and Reproduction. Front. Immunol..

[118] Gibbs A., Leeansyah E., Introini A., Paquin-Proulx D., Hasselrot K., Andersson E., Broliden K., Sandberg J.K., Tjernlund A. (2017). MAIT cells reside in the female genital mucosa and are biased towards IL-17 and IL-22 production in response to bacterial stimulation. Mucosal Immunol..

[119] Lund J.M., Hladik F., Prlic M. (2023). Advances and challenges in studying the tissue-resident T cell compartment in the human female reproductive tract. Immunol. Rev..

[120] Prašnikar E., Kunej T., Gorenjak M., Potočnik U., Kovačič B., Knez J. (2022). Transcriptomics of receptive endometrium in women with sonographic features of adenomyosis. Reprod. Biol. Endocrinol..

[121] Jewanraj J., Ngcapu S., Osman F., Mtshali A., Singh R., Mansoor L.E., Abdool Karim S.S., Abdool Karim Q., Passmore J.S., Liebenberg L.J.P. (2020). The Impact of Semen Exposure on the Immune and Microbial Environments of the Female Genital Tract. Front. Reprod. Health.

[122] Koga K., Izumi G., Mor G., Fujii T., Osuga Y. (2014). Toll-like receptors at the maternal-fetal interface in normal pregnancy and pregnancy complications. Am. J. Reprod. Immunol..

[123] Benjelloun F., Quillay H., Cannou C., Marlin R., Madec Y., Fernandez H., Chrétien F., Le Grand R., Barré-Sinoussi F., Nugeyre M.T. (2020). Activation of Toll-Like Receptors Differentially Modulates Inflammation in the Human Reproductive Tract: Preliminary Findings. Front. Immunol..

[124] Cuadrado-Torroglosa I., García-Velasco J.A., Alecsandru D. (2024). The Impacts of Inflammatory and Autoimmune Conditions on the Endometrium and Reproductive Outcomes. J. Clin. Med..

[125] Gholiof M., Adamson-De Luca E., Wessels J.M. (2022). The female reproductive tract microbiotas, inflammation, and gynecological conditions. Front. Reprod. Health.

[126] Berryman M.A., Ilonen J., Triplett E.W., Ludvigsson J. (2023). Important denominator between autoimmune comorbidities: A review of class II HLA, autoimmune disease, and the gut. Front. Immunol..

[127] Ludgate M.E., Masetti G., Soares P. (2024). The relationship between the gut microbiota and thyroid disorders. Nat. Rev. Endocrinol..

[128] Godines-Enriquez M.S., Miranda-Velásquez S., Enríquez-Pérez M.M., Arce-Sánchez L., Martínez-Cruz N., Flores-Robles C.M., Aguayo-González P., Morales-Hernández F.V., Villarreal-Barranca A., Suárez-Rico B.V. (2021). Prevalence of Thyroid Autoimmunity in Women with Recurrent Pregnancy Loss. Medicina.

[129] Turesheva A., Aimagambetova G., Ukybassova T., Marat A., Kanabekova P., Kaldygulova L., Amanzholkyzy A., Ryzhkova S., Nogay A., Khamidullina Z. (2023). Recurrent Pregnancy Loss Etiology, Risk Factors, Diagnosis, and Management. Fresh Look into a Full Box. J. Clin. Med..

[130] Buendia-Roldan I., Ponce-Gallegos M.A., Lara-Beltrán D., Del Ángel-Pablo A.D., Pérez-Rubio G., Mejía M., Selman M., Falfán-Valencia R. (2022). The HLA-DRB1*07 Allele Is Associated with Interstitial Lung Abnormalities (ILA) and Subpleural Location in a Mexican Mestizo Population. Biomolecules.

[131] Miko E., Barakonyi A. (2023). The Role of Hydrogen-Peroxide (H_2_O_2_) Produced by Vaginal Microbiota in Female Reproductive Health. Antioxidants.

[132] Vanstokstraeten R., Callewaert E., Blotwijk S., Rombauts E., Crombé F., Emmerechts K., Soetens O., Vandoorslaer K., De Geyter D., Allonsius C. (2023). Comparing Vaginal and Endometrial Microbiota Using Culturomics: Proof of Concept. Int. J. Mol. Sci..

[133] Osadchiy V., Belarmino A., Kianian R., Sigalos J.T., Ancira J.S., Kanie T., Mangum S.F., Tipton C.D., Hsieh T.-C.M., Mills J.N. (2024). Semen microbiota are dramatically altered in men with abnormal sperm parameters. Sci. Rep..

[134] Doroftei B., Ilie O.D., Armeanu T., Stoian I.L., Anton N., Babici R.G., Ilea C. (2023). A Narrative Review Discussing the Obstetric Repercussions Due to Alterations of Personalized Bacterial Sites Developed within the Vagina, Cervix, and Endometrium. J. Clin. Med..

[135] Faught B.M., Reyes S. (2019). Characterization and Treatment of Recurrent Bacterial Vaginosis. J. Women’s Health.

[136] Rahman N., Mian M.F., Nazli A., Kaushic C. (2023). Human vaginal microbiota colonization is regulated by female sex hormones in a mouse model. Front. Cell. Infect. Microbiol..

[137] Shen J., Song N., Williams C.J., Brown C.J., Yan Z., Xu C., Forney L.J. (2016). Effects of low dose estrogen therapy on the vaginal microbiomes of women with atrophic vaginitis. Sci. Rep..

[138] Gustin A.T., Thurman A.R., Chandra N., Schifanella L., Alcaide M., Fichorova R., Doncel G.F., Gale M., Klatt N.R. (2022). Recurrent bacterial vaginosis following metronidazole treatment is associated with microbiota richness at diagnosis. Am. J. Obstet. Gynecol..

[139] Tuniyazi M., Zhang N. (2023). Possible Therapeutic Mechanisms and Future Perspectives of Vaginal Microbiota Transplantation. Microorganisms.

[140] Meng Y., Sun J., Zhang G. (2024). Vaginal microbiota transplantation is a truly opulent and promising edge: Fully grasp its potential. Front. Cell. Infect. Microbiol..

[141] Martinelli S., Nannini G., Cianchi F., Staderini F., Coratti F., Amedei A. (2023). Microbiota Transplant and Gynecological Disorders: The Bridge between Present and Future Treatments. Microorganisms.

[142] Wrønding T., Vomstein K., Bosma E.F., Mortensen B., Westh H., Heintz J.E., Mollerup S., Petersen A.M., Ensign L.M., DeLong K. (2023). Antibiotic-free vaginal microbiota transplant with donor engraftment, dysbiosis resolution and live birth after recurrent pregnancy loss: A proof of concept case study. eClinicalMedicine.

[143] Lyra A., Ala-Jaakkola R., Yeung N., Datta N., Evans K., Hibberd A., Lehtinen M.J., Forssten S.D., Ibarra A., Pesonen T. (2023). A Healthy Vaginal Microbiota Remains Stable during Oral Probiotic Supplementation: A Randomised Controlled Trial. Microorganisms.

[144] Husain S., Allotey J., Drymoussi Z., Wilks M., Fernandez-Felix B.M., Whiley A., Dodds J., Thangaratinam S., McCourt C., Prosdocimi E.M. (2020). Effects of oral probiotic supplements on vaginal microbiota during pregnancy: A randomised, double-blind, placebo-controlled trial with microbiome analysis. BJOG.

[145] Marcotte H., Larsson P.G., Andersen K.K., Zuo F., Mikkelsen L.S., Brandsborg E., Gray G., Laher F., Otwombe K. (2019). An exploratory pilot study evaluating the supplementation of standard antibiotic therapy with probiotic lactobacilli in south African women with bacterial vaginosis. BMC Infect. Dis..

[146] Rafiee M., Sereshki N., Alipour R., Ahmadipanah V., Pashoutan Sarvar D., Wilkinson D. (2022). The effect of probiotics on immunogenicity of spermatozoa in couples suffering from recurrent spontaneous abortion. BMC Immunol..

[147] Giannella L., Grelloni C., Quintili D., Fiorelli A., Montironi R., Alia S., Delli Carpini G., Di Giuseppe J., Vignini A., Ciavattini A. (2023). Microbiome Changes in Pregnancy Disorders. Antioxidants.

[148] Qi F., Fan S., Fang C., Ge L., Lyu J., Huang Z., Zhao S., Zou Y., Huang L., Liu X. (2023). Orally administrated *Lactobacillus gasseri* TM13 and Lactobacillus crispatus LG55 can restore the vaginal health of patients recovering from bacterial vaginosis. Front. Immunol..

[149] Hertz F.B., Holm J.B., Pallejá A., Björnsdóttir M.K., Mikkelsen L.S., Brandsborg E., Frimodt-Møller N. (2022). Vaginal microbiome following orally administered probiotic. APMIS.

[150] Li Z., Zheng Y., Zhang M., Wu K., Zhang L., Yao Y., Zheng C. (2024). Gut microbiota-derived metabolites associate with circulating immune cell subsets in unexplained recurrent spontaneous abortion. Heliyon.

[151] Zargar M., Ghafourian M., Behrahi F., Nikbakht R., Salehi A.M. (2024). Association of recurrent implantation failure and recurrent pregnancy loss with peripheral blood natural killer cells and interferon-gamma level. Obstet. Gynecol. Sci..

[152] Wang W., Zhou X., Zhang Y., Chen Z., Huang J., Zhang X., Kwak-Kim J. (2022). The characteristics of antigenic specificity of memory regulatory t cells in women with unexplained recurrent pregnancy loss. J. Reprod. Immunol..

[153] Tian Z., Zhang X., Yao G., Jin J., Zhang T., Sun C., Wang Z., Zhang Q. (2024). Intestinal flora and pregnancy complications: Current insights and future prospects. iMeta.

[154] .Lu X., Shi Z., Jiang L., Zhang S. (2024). Maternal gut microbiota in the health of mothers and offspring: From the perspective of immunology. Front. Immunol..

[155] Esparvarinha M., Madadi S., Aslanian-Kalkhoran L., Nickho H., Dolati S., Pia H., Danaii S., Taghavi S., Yousefi M. (2023). Dominant immune cells in pregnancy and pregnancy complications: T helper cells (TH1/TH2, TH17/Treg cells), NK cells, MDSCs, and the immune checkpoints. Cell Biol. Intern..

[156] Jin M., Li D., Ji R., Liu W., Xu X., Feng X. (2020). Changes in Gut Microorganism in Patients with Positive Immune Antibody-associated Recurrent Abortion. BioMed. Res. Int..

[157] Zhang L., Li Q., Su Y., Zhang X., Qu J., Liao D., Zou Q., Zou H., Liu X., Li C. (2023). Proteomic profiling analysis of human endometrium in women with unexplained recurrent spontaneous abortion. J. Proteom..

[158] Ali S., Majid S., Ali M.N., Taking S., Rehman M.U., Arafah A. (2021). Cytokine imbalance at the materno-embryonic interface as a potential immune mechanism for recurrent pregnancy loss. Int. Immunopharmacol..

[159] Dingle K., Kassem O.M., Azizieh F., AbdulHussain G., Raghupathy R. (2023). Quantitative analyses of cytokine profiles reveal hormone-mediated modulation of cytokine profiles in recurrent spontaneous miscarriage. Cytokine.

[160] Mazziotta C., Tognon M., Martini F., Torreggiani E., Rotondo J.C. (2023). Probiotics Mechanism of Action on Immune Cells and Beneficial Effects on Human Health. Cells.

[161] Virk M.S., Virk M.A., He Y., Tufail T., Gul M., Qayum A., Rehman A., Rashid A., Ekumah J.-N., Han X. (2024). The Anti-Inflammatory and Curative Exponent of Probiotics: A Comprehensive and Authentic Ingredient for the Sustained Functioning of Major Human Organs. Nutrients.

[162] Di Pierro F., Sinatra F., Cester M., Da Ros L., Pistolato M., Da Parè V., Fabbro L., Maccari D., Dotto S., Sossai S. (2023). Effect of *L. crispatus* M247 Administration on Pregnancy Outcomes in Women Undergoing IVF: A Controlled, Retrospective, Observational, and Open-Label Study. Microorganisms.

[163] Barati M., Jabbari M., Ghavidel A.A., Nikmehr P., Arzhang P., Aynehchi A., Babashahi M., Mosharkesh E., Roshanravan N., Shabani M. (2022). The engineered probiotics for the treatment of chronic diseases: A systematic review. J. Food Biochem..

[164] Kemp M.W., Newnham J.P., Challis J.G., Jobe A.H., Stock S.J. (2016). The clinical use of corticosteroids in pregnancy. Hum. Reprod. Update.

[165] Giulini S., Grisendi V., Sighinolfi G., Di Vinci P., Tagliasacchi D., Botticelli L., La Marca A., Facchinetti F. (2022). Chronic endometritis in recurrent implantation failure: Use of prednisone and IVF outcome. J. Reprod. Immunol..

[166] Hart R.J. (2023). Nutritional supplements and IVF: An evidence-based approach. Reprod. Biomed. Online.

[167] Piekarska K., Dratwa M., Radwan P., Radwan M., Bogunia-Kubik K., Nowak I. (2023). Pro- and anti-inflammatory cytokines and growth factors in patients undergoing in vitro fertilization procedure treated with prednisone. Front. Immunol..

[168] Su Q., Pan Z., Yin R., Li X. (2024). The value of G-CSF in women experienced at least one implantation failure: A systematic review and meta-analysis. Front. Endocrinol..

[169] Yao K., Sun Y., Ye X., Wu Y. (2023). Interferon-λ contributes to endometrial receptivity. Reproduction.

[170] Nnamonu E.I., Mgbenka B.O., Mbegbu E.C. (2020). Impact of omega-3 fatty acids preconception intake on some fertility parameters and foetuses quality of female rats. Iran. J. Vet. Res..

[171] Trop-Steinberg S., Gal M., Azar Y., Kilav-Levin R., Heifetz E.M. (2024). Effect of omega-3 supplements or diets on fertility in women: A meta-analysis. Heliyon.

[172] Mu F., Huo H., Wang M., Wang F. (2023). Omega-3 fatty acid supplements and recurrent miscarriage: A perspective on potential mechanisms and clinical evidence. Food Sci. Nutr..

[173] Kello N., Cho Y.M. (2024). Natural supplements in antiphospholipid syndrome: A case for further study. Clin. Immunol..

[174] Chen P., Yang M., Chen R., Chen P., Chen L., Fang C., Li T. (2023). Endometrial microbial alterations disrupt endometrial immune homeostasis by overactivation of Eicosapentaenoic acid biosynthesis leading to altered endometrial receptivity. J. Reprod. Immunol..

[175] Izadifar Z., Cotton J., Chen S., Horvath V., Stejskalova A., Gulati A., LoGrande N.T., Budnik B., Shahriar S., Doherty E.R. (2024). Mucus production, host-microbiome interactions, hormone sensitivity, and innate immune responses modeled in human cervix chips. Nat. Commun..

